# Allometric scale model reveals temperature effects on growth and reproduction in *Daphnia magna*

**DOI:** 10.1038/s41598-025-15593-6

**Published:** 2025-08-22

**Authors:** Hideyasu Shimadzu, Miguel Barbosa

**Affiliations:** 1 Department of Data Science, Kitasato University, Kanagawa, Japan; 2 Centre for Biological Diversity, School of Biology, University of St Andrews, Fife, UK; 3 Department of Biology, University of Aveiro, Aveiro, Portugal

**Keywords:** Differential equations, Power law, Statistical methods, Climate-change ecology, Freshwater ecology

## Abstract

Climate change amplifies temperature variability, thereby subjecting organisms to increased stress as they more frequently encounter temperatures outside their optimal range. Temperature influences resource distribution across fundamental processes in organisms, such as metabolism, reproduction and overall fitness, yet energy allocation strategies are primarily understood under stable temperature conditions. Predicting organisms’ responses to fluctuating temperatures, however, remains challenging. To address this gap, we develop an allometric growth model to predict energy allocation between growth and reproduction under both constant and variable temperature conditions. The model predictions perform and align well with the observed growth patterns of *Daphnia magna*, a keystone species in aquatic ecosystems, exposed to various thermal scenarios. Results indicate that exposure to unpredictable temperatures elicits growth and reproduction responses similar to those observed under consistently high temperatures. However, individuals exposed to unpredictable temperatures incur a disproportionate energetic cost compared to those in constant average or low-temperature conditions, significantly reducing estimated fecundity over time and lifespan. These findings highlight the relative energetic impacts of increased unpredictability in temperature and underline its critical role in shaping life-history traits. Given the growing concern over modern climate change scenarios, the allometric growth model provides a straightforward yet essential approach for integrating energetic and subsequent ecological effects, enabling not only predictions of responses across keystone species but also an enhanced understanding of anthropogenic impacts on aquatic ecosystems.

## Introduction

Temperature plays a fundamental role in shaping resource allocation patterns by influencing metabolic demands, energy budgets and trade-offs between growth, reproduction and maintenance^1^. As temperature increases, metabolic rates typically rise^2^, leading to greater energy expenditure on maintenance and activity at the expense of growth and reproductive investment. This shift often results in reduced body size, a phenomenon known as the temperature-size rule, where individuals mature earlier but at smaller sizes^3^. Further, warmer temperatures can favour increased reproductive output in some species, while others may experience constraints due to elevated metabolic costs. In contrast, colder environments often promote energy storage strategies, with greater allocation to lipid reserves to enhance survival during resource-scarce periods^1^. Temperature-driven shifts in resource allocation underpins responses in life-history traits^4^ which have significant ecological and evolutionary implications, influencing population dynamics, species interactions, and overall ecosystem function, particularly in the face of climate change^5^.

Species often respond to predictable changes in mean temperature by adjusting the reproductive cycles and brood sizes to maximise fitness^6^. However, responses to increased temperature variability and its associated unpredictability introduce further challenges nonetheless^7^. Although life-history responses to predictable constant temperatures are well-studied and understood, our comprehension remains comparatively limited regarding the effects of unpredictable fluctuation in temperature on energy allocation—and the subsequent trade-offs between reproduction, growth and lifespan^8,9^. Further, there is no unifying agreement regarding the fitness effects of increase unpredictability, with both positive and negative impacts being reported^10^. In this study, we address this scientific gap by developing a novel temperature-energy model that quantifies the fitness (reproduction), growth and mortality costs associated with the increased temperature unpredictability forecasted under modern climate change scenarios.

Mathematical models play a pivotal role in comprehending the energy allocation between growth and reproduction, often using surrogate metrics, such as body mass, length and reproduction data^11^. Energy expenditure on growth and reproduction has been expressed through differential equations rooted in the von Bertalanffy-Pütter differential equation^12–14^, which specifies growth as a balance between anabolic and catabolic processes. To date, the subsequent model advancements have shifted towards increasingly mechanistic formulations, offering greater biophysical interpretability through parameters, which align with underlying metabolic processes rather than relying solely on phenomenological observations. A key aspect is model exponents, which both distinguish the theoretical underpinnings of model developments (see Kearney 2020^15^ for a comprehensive review) and ensure that the differential equation possesses an analytical solution whereby model parameters can be estimated using body length data.

Previous major model advancements include the Dynamic Energy Budget (DEB) theory^16,17^, which expresses a rate parameter at which energy assimilation and partition between reproduction, growth and maintenance are specified and related to environmental conditions with organism-specific parameters^16–18^. The DEB framework has been widely employed to capture the energetic effect of various stressors, including chemical, temperature and food-related factors, on the life-history of freshwater organisms^19^. In principle, the DEB model differs from the von Bertalanffy-Pütter model, specifying the model terms as assimilation and maintenance costs encompassing anabolic and catabolic processes. This distinction is apparent in the core parameters, which differ depending upon the theoretical framework on which each model stands^15^. Under constant temperature and resources (*i.e.* food), the growth curve proposed by the DEB coincides with the von Bertalanffy growth curve on two parameters: (1) the relative growth rate coefficient and (2) the structural length^18,20^. While these two parameters can be estimated from longitudinal body length data, these are also confounded regarding metabolic aspects by more than two core parameters. Thus, all these core parameters can be mathematically unidentifiable unless some of the parameters are predetermined or estimated from some additional sources. This fact indicates an alternative model refinement, leading to the alteration of the von Bertalanffy-Pütter differential equation, which may incline towards a hybrid model that combine mechanistic and phenomenological approaches, simplifying model parameters while still maintaining meaningful biophysical interpretations.

There is some recognised limitations of using the von Bertalanffy-Pütter equation to estimate the energy component allocated to reproduction^21^. However, recent model advancements have integrated the energy component allocated to reproduction to be a feature of the allometric scaling model framework^22–25^. Here, we develop a novel allometric scaling model to capture individual lifetime energy allocation shifts between growth and non-growth components. Model parameters are validated using *D. magna* exposed to different temperature scenarios (*i.e.* constant-low, constant-rearing, constant-high and unpredictable temperatures). Combined with our experiments, the proposed model offers several important scientific contributions. First, the model introduces a time-varying rate parameter at which acquired energy is partitioned between growth and reproduction components, illustrating age-dependent transitions. Second, all the model parameters, including the exponents typically predefined in other modelling approaches, are estimable from body length data alone, also bypassing the need of solving the original differential equation. This flexibility enables the model to delineate energy allocated to reproduction without fecundity data, broadening its applicability across species and systems with similar biological traits to our study organism. Importantly, these extensions are achieved by model re-parametrisation accommodating the present experiment design. Finally, our model reveals a more realistic insight into the energy allocation and bioenergetic costs incurred throughout their lifetime under different temperature scenarios, rather than simply studying short or chronic exposures to stress, shedding light on the extent to which species may respond to increasingly unpredictable anthropogenic climate change.

## Data and model

### Experiment and data

All the individuals used in the experiment were descendants ($\hbox {F}_1$) from the third brood neonates of *D. magna* clone F^26^. The parental generation ($\hbox {F}_0$) was raised at a constant temperature of 20$^\circ$C under a 16:8-hour (light:dark) photoperiod in ASTM (American Society for Testing Materials) medium^27^ and individually fed daily with green algae, *Pseudokirchneriella subcapitata*, at a concentration of $3.0 \times 10^5$ cells $\hbox {ml}^{-1}$ (see ASTM 1980^28^). This concentration was above *ad libitum* to ensure proportional satiation of energetic requirements across all temperature scenarios. The medium was changed every other day. The temperature, photoperiod and feeding rate used in our cultures follow guideline 211 from the Organization for Economic and Co-operation and Development, which is recommended for reproduction and chemical tests with *Daphnia*^27^.

Each $\hbox {F}_1$ individual was, immediately after birth, randomly placed in an individual 50 ml glass container and allocated to one of four temperature scenarios over their entire life and maintained within a Binder incubator (Binder Bs28). The four temperature scenarios were: constant low (15$^\circ$C), constant rearing (20$^\circ$C), constant high (25$^\circ$C), and unpredictable variation (15–25$^\circ$C, see the supplementary information). The constant rearing temperature refers to the *normal* temperature at which individuals are kept in the laboratory. The maximum temperature was set at 25$^\circ$C. This value is above the optimal temperature curve for *D. magna*, thus expected to elicit oxidative stress which will impact growth, reproduction and survival^29,30^. Under the unpredictable temperature scenario, temperature randomly fluctuated but within different ranges according to three specific time segments every day. From 00:00 to 08:00 and 18:00 to 24:00 (dawn–morning and late afternoon segments), the temperature fluctuated within 15–20$^\circ$C; from 08:00 to 18:00 (morning–afternoon segment), it randomly varied within 20–25$^\circ$C. This resulted in an overall average of 19.8$^\circ$C, matching the constant rearing temperature. Thus, any deviation in observed growth and reproduction patterns under the unpredictable temperature scenario should be attributed to temperature variability. We obtained data from 628 $\hbox {F}_1$ individuals (high: 157; rearing: 156; low: 158; and unpredictable: 157), each fed as per $\hbox {F}_0$ protocol, with the culture medium changed every two days. By providing the same amount of food, we can better isolate the temperature effect because of the fixed amount of energy intake being allocated to growth ($\kappa$) and reproduction ($1-\kappa$), according to the DEB framework.

We measured $\hbox {F}_1$ body length, $\{l_{it}\}, t=0, t_1, t_2, \ldots , t_{k_i}$, for every individual *i* upon the birth of neonates ($\hbox {F}_2$), $t=t_1, t_2, \ldots , t_{k_i-1}$; we also recorded $\hbox {F}_1$ body length at their birth, $t=0$, and death, $t=t_{k_i}$. Time of birth of neonates was considered as the time point when neonates were released and observed in the vial. Note that the time intervals between body length observations are thus uneven, unlike typical time-series data, which will be considered when fitting the model. Each $\hbox {F}_1$ individual was placed in a culture cell plate using a 3 ml plastic pipette and photographed to measure the body length (from the tip of the head to the start of the caudal spine) to the nearest micrometres using ImageJ software^31^ (ver. 1.54g; https://imagej.net/ij/index.html). In addition, we recorded the number of $\hbox {F}_2$ individuals, $\{ n_{it}\}$, for each brood, $t=t_1, t_2, \ldots , t_{k_i-1}$. The experiment was continued until the last $\hbox {F}_1$ individual died. We recorded, immediately after emergence, more than 130,000 neonates across all four scenarios.

### Modelling

Under specific assumptions body mass can be used to estimate patterns of growth rate under variants of Pütter equation^12^ that include the von Bertalanffy^13^, the Gompertz^32^ and other logistic growth models as special cases. However, body mass can be converted to body length, another common unit, under some assumptions. There is some variability in how body length scales with mass, however, most studies on *Daphnia* suggest using a cubic transformation^33–35^. The growth model here combines the knowledge from the DEB theory and a recently proposed modelling framework^25^, and its extension hinges upon two aspects: 1) the model introduces a time-varying rate parameter, later denoted as $r_t$, at which acquired energy is partitioned into growth and reproduction components over a lifetime; and 2) all the model parameters, including the time-varying rate parameter, can be estimated solely from body length data. The new model discussed here is constructed in two steps. First, the model is developed and specified as a general form based on body mass. Second, the proposed body mass model is then converted into a unit of length.

#### Body mass model

We consider the following differential equations to describe individual body mass $m_t$ at age *t*, which is made up of the somatic $s_t$ and gonadic $g_t$ masses, in allometric forms *viz.*

1a $$\begin{aligned} dm_t= & ds_t + dg_t, \end{aligned}$$

1b $$\begin{aligned} ds_t= & a s_t^\alpha dt - c s_t^\gamma dt - dg_t, \end{aligned}$$

1c $$\begin{aligned} dg_t= & bs_t^\beta dt. \end{aligned}$$

We assume that the gonadic mass is zero, $g_t=0$, at times of the individual’s birth ($t=0$) and of $\hbox {F}_2$ neonates’ birth ($t=t_1, t_2, \ldots , t_{k-1}$). In standard allometric equations, each model term comprises somatic mass $s_t$ and two parameters: the exponent (*i.e.* $\alpha , \beta$ and $\gamma$) that describes how the parameter scales over different values of body mass; and the multiplier (*i.e.* *a*, *b* and *c*) that often refers to a parameter independent of body mass. Eq. (1a) states a constraint that the somatic and gonadic masses must be the total mass. Equations (1b)–(1c) describe the energy allocation mechanism of individuals. Equation (1b) consists of three components: $a s_t^\alpha$ expresses the acquisition of energy resources; $c s_t^\gamma$ describes energy expenditure towards non-body-growth components, including maintenance and indirect reproduction costs. The term $dg_t$ then represents the direct reproduction energy expenditure that is specified in Eq. (1c). The model here assumes that investments in gonadic growth (1c) begin immediately after birth. Some allometric models often assume a two-stage response where energy is allocated to gonadic growth after a certain age of maturity^22,23,25^. Such energetic allocation response is advantageous for species that take longer to reach sexual maturity (*e.g.* fish and mammals). There is also a direct link to other growth models; for example, von Bertalanffy model assumes specific constants for the exponent as $\alpha =2/3, \gamma =1$ but does not explicitly accommodate the gonadic energy allocation $dg_t$ (see Ricklefs (2003)^36^ for mathematical links to other models). The key distinction here is the model exponents that are not predefined but estimated from data, which makes the present model hybrid contrasting to those existing models.

Given the sum-constraint (1a), we can write the instantaneous relative fecundity investment, $r_t$ say, taking the ratio of Eqs. (1c) and (1a) as

2 $$\begin{aligned} r_t = \frac{dg_t}{dm_t} = \frac{b}{a(1-p_t)} s_t^{\beta -\alpha }, \end{aligned}$$

where $p_t=c s_t^{\gamma -\alpha }/a$, the rate at which energy is allocated to non-body-growth components. The rate $r_t$ can take, from the definition, values between $0 \le r_t <1$. Given this, we can re-write Eq. (2) as

3 $$\begin{aligned} b s_t^{\beta } =a (1-p_t) r_t s_t^{\alpha }, \end{aligned}$$

and substitute it to Eq. (1b) as

1b’ $$\begin{aligned} ds_t = a (1-p_t) (1-r_t) s_t^\alpha dt. \end{aligned}$$

The analytical solution of Equation $(\hbox {1b}^{\prime })$ cannot be obtained in a closed form. Thus, numerical means is required to solve the equation or estimate the model parameters.

#### Body length model

Once the growth model is established in terms of body mass, we can scale it into a unit of body length. Somatic mass $s_t$ and body length $l_t$ are scaled using a power transformation,

4 $$\begin{aligned} s_t = h l_t^k, \end{aligned}$$

where *h* and *k* are respectively a constant. Since $dl^k_t/dt=k l_t^{k-1}(dl_t/dt)$, the conversion emerges to a simple re-parametrisation of Equation $(\hbox {1b}^{\prime })$ as

5 $$\begin{aligned} dl_t = z (1 - p_t) (1-r_t ) l_t^{\lambda } dt, \end{aligned}$$

where

$$\begin{aligned} z = \frac{a}{k}h^{\alpha -1}; ~ p_t=\frac{c}{a} \left( h l_t^k \right) ^{\gamma -\alpha }; ~ \lambda =k(\alpha -1)+1~\textrm{and}~ r_t = \frac{dg_t}{dm_t}. \end{aligned}$$

The above re-parametrisation inherits the implication from the original parameters of the body mass model (1b). The parameters *z* and $\lambda$ only depend on the energy acquisition through the parameters *a* and $\alpha$, whereas the time-varying parameter $p_t$ relies on both the energy acquisition and the non-growth (i.e. maintenance and indirect reproduction) costs through the parameters *c* and $\gamma$, apart from the constant *h* used for the body mass–length transformation (Eq. 4). If it is assumed that $\alpha =\gamma$, *i.e.* the energy allocated to the non-growth components is proportional to the energy intake as $c(a s_t^\alpha )$, then $p_t$ becomes constant over time as $p=c/a$.

#### Lifetime fecundity model

Equation (1c) suggests that the cumulative energy allocated to direct reproduction up to age $\tau$ can be quantified by its integration. From Eqs. (3) and (4), this quantity is given as

6 $$\begin{aligned} g_\tau = \int _0^{\tau } dg_t = \int _0^{\tau } a (1-p_u) r_u s_u^{\alpha } du = \int _0^{\tau } z (1-p_u) r_u l_u^{\lambda } du. \end{aligned}$$

Total energy allocated to reproduction is difficult to quantify directly from standard experiments. Here we use the cumulative number of neonates produced by each individual up to age $\tau$, say $N_\tau = \sum _{t \le \tau } n_t$, to capture the energy allocated to direct reproduction. Accordingly, we expect the cumulative number of neonates produced across the lifetime to be proportional to the cumulative energy, $N_\tau \propto g_\tau$, assuming that the energy required per neonate is relatively constant. In *Daphnia*, the primary energy source for reproduction is provided by lipids obtained through food^37^. Although the amount of triacylglycerol transferred into each egg depends on age and feeding success, within the individual, the energy cost of producing a neonate is expected to be similar^38^.

### The model assumptions for the present experiment

The growth model *sensu* body length is provided in a general form (Eq. 5), which can be re-parametrised to reflect the experimental conditions used here. This facilitates the interpretation of what each model parameter represents in energy allocation mechanisms. We set the following three assumptions:

1. the potential energy intake from food is identical for all individuals since all individuals were fed above *ad libitum* in a controlled feeding environment ensuring that nutritional adequacy. Thus the parameters *z* and $\lambda$ of Eq. (5) will become the same over the different temperature scenarios. There is a similar convergence in the parameters *a* and $\alpha$ of Equation $(\hbox {1b}^{\prime })$ in the body mass context;
2. the amount of energy spent on maintenance is proportional to the energy intake^18,37^. The parameter $p_t$ of Equation (5) is thus constant over a lifetime but can differ amongst the temperature scenarios; and
3. the amount of energy allocated to direct reproduction differs amongst the different temperature scenarios^35^. Accordingly, the time-varying parameter $r_t$ of Eq. (5) will depend upon time and temperature scenarios.

Under these assumptions, Eq. (5) can be re-parametrised *viz.*

7 $$\begin{aligned} dl_t = q(1-r_t) l_t^{\lambda } dt, \end{aligned}$$

with two constant parameters, *q* and $\lambda$ and a time-varying parameter, $r_t$, that are

7a $$\begin{aligned} q= & \frac{h^{\alpha -1}}{k}(a-c); \end{aligned}$$

7b $$\begin{aligned} \lambda= & k(\alpha -1)+1 ~ \textrm{and} \end{aligned}$$

7c $$\begin{aligned} r_t= & \frac{dg_t}{dm_t}. \end{aligned}$$

To specify Eq. (7), these parameters, $q, \lambda$ and $r_t$ (see Table 1 for their interpretation), need to be estimated from body length data. It is worthwhile mentioning that our model advancement allows, as will be discussed in the following section, estimating the time-varying parameter $r_t$ without body ($m_t$) and gonadic ($g_t$) mass data, both of which are initially required as the model states.

**Table 1:** The key model parameters in Eq. 7.

Parameter	Description	Eq
*q*	The scalar indicating the energy portion allocated to body growth.	7a
$\lambda$	The scale exponent of body length (equivalent to $\alpha$ in body mass, Eq. 1b).	7b
$r_t$	The proportion of energy invested towards fecundity.	7c

The assumptions and the re-parametrisation above will benefit model interpretation. Differences in energy allocation patterns caused by different temperature scenarios can now be revealed *via* the parameters *q* and $r_t$. Since the parameters *a* and $\alpha$ are related to anabolism and are assumed to be the same across the temperature scenarios (A1), the parameter *q* tends to be small when energy costs become greater because of a larger value of *c*; note that $\alpha =\gamma$ (A2). In other words, individuals are predicted to grow slowly when values of *q* are small because of less energy for growth. The extent to which energy is allocated to direct reproduction changes over time across the different temperature scenarios is given by the time-varying parameter $r_t$ (A3). However, as Eq. (7) cannot be solved analytically, the parameter estimation relies on numerical means.

Once the model parameters, $q, \lambda$, and $r_t$ in Eq. (7) are estimated from body length data, the total energy allocated to direct reproduction up to age $\tau$ should mirror the pattern of the cumulative number of neonates up to age $\tau$, say $N_\tau = \sum _{t \le \tau } n_t$. This allows us to test whether the estimated model represents the actual energy allocation mechanism well by evaluating the shape of the curve against the number of neonates produced by each individual over a lifetime. The expected number of notates up to age $\tau$ can be proportional to the cumulative energy, $g_\tau$, as

8 $$\begin{aligned} N_\tau \propto q \int _0^{\tau } r_u l_u^{\lambda } du. \end{aligned}$$

### Parameter estimation

The parameter estimation procedure employed the gradient matching method to minimise the squared errors in the gradient (derivative) without solving the differential equation^25,39–41^. The overview of the parameter estimation procedure is provided below. Note that the hat-sign ( $\hat{\cdot }$ ) is hereafter used for variables and parameters throughout the manuscript to indicate *estimated* variable or parameter values.

Consider observed body length trajectory data $\{l_{it} \}$ for the *i*-th individual. Time *t* takes discrete time points, $t=0, t_1, \ldots , t_{k_i}$, and the endpoint $t_{k_i}$ differs amongst individuals because their lifetime varies. The observed trajectories are noisy realisations from the process $l_t$ governed by the differential Eq. (7). The observations can then be written as

9 $$\begin{aligned} l_{it} = \hat{l}_{it} + \varepsilon _{it}, \end{aligned}$$

where $\varepsilon _{it}$ is a noise term with mean of zero, $\mathbb {E}\left[ {\varepsilon _{it}}\right] =0$ for $t=0, t_1, \ldots , t_{k_i}$. Although the analytical form of the processes (Eq. 7) are unknown, Eq. (9) suggests that the form can be delineated from the data, taking their expectation, *i.e.* $\hat{l}_{it} {:}{=} \mathbb {E}\left[ {l_{it}}\right]$. The calculation of the expectation here is carried out *via* the locally weighted regression (loess)^42,43^ as described in Shimadzu & Wang (2021)^25^ with the default smoothing argument.

The stochastic version of the differential Eq. (7) can be described in a conventional way *viz.*

10 $$\begin{aligned} d\hat{l}_{it} = q \left( 1-r_t \right) \hat{l}_{it}^{\lambda } dt + \sigma dB_{it}. \end{aligned}$$

The standard Brownian motion $B_{it}$, with its mean $\mathbb {E}\left[ {dB_{it}}\right] =0$, describes stochastic divergence from the model due to the stochastic nature amongst time and individuals. This discrepancy is going to be minimised when estimating the parameters, namely *q*, $\lambda$ and $r_t$ as

$$\begin{aligned} (\hat{q}, \hat{\lambda }, \hat{r}_t)= & \arg \min \sum _{i} \int \left\{ \frac{d \hat{l}_{it}}{dt} - q \left( 1-r_t \right) \hat{l}_{it}^{\lambda } \right\} ^2 dt, \\\approx & \arg \min \sum _{i} \sum _{t=0}^{t_{k_i}} w_t \left\{ \frac{d \hat{l}_{it}}{dt} - q \left( 1-r_t \right) \hat{l}_{it}^{\lambda } \right\} ^2. \end{aligned}$$

The integrand above is a squared term of the stochastic divergence specified in Eq. (10). To estimate the approximate value of the above integral, we use the trapezium method with appropriate intervals; $w_t$ is a weight for the numerical integration, and the present study chooses $dt=0.5$ (day). It is worth noting that the minimisation procedure here is the same as the weighted least squared method for linear regressions, profiling upon the parameter $\lambda$—profile least squares—which can easily be implemented in a standard linear regression framework.

## Results

**Table 2:** The parameter estimates and summary statistics with their $95\%$ confidence intervals.

Treatments	$\hat{q}$	$\hat{\lambda }^{\dagger }$	$\hat{N}_\tau$	$\hat{\tau }$
	(Eq. 7a)	(Eq. 7b)		
Constant Low	0.118		239	81
(15$^\circ$C)	(0.110, 0.126)		(235, 243)	(75, 87)
Constant Rearing	0.169	0.179	236	72
(20$^\circ$C)	(0.159, 0.180)	(0.163, 0.196)	(229, 242)	(67, 77)
Constant High	0.209		181	48
(25$^\circ$C)	(0.198, 0.220)		(176, 185)	(45, 52)
Unpredictable Variation	0.196		194	52
(15–25$^\circ$C)	(0.187, 0.206)		(189, 199)	(48, 56)

$\dagger$: The estimate is assumed to be the same over the different treatments

**Fig. 1:**
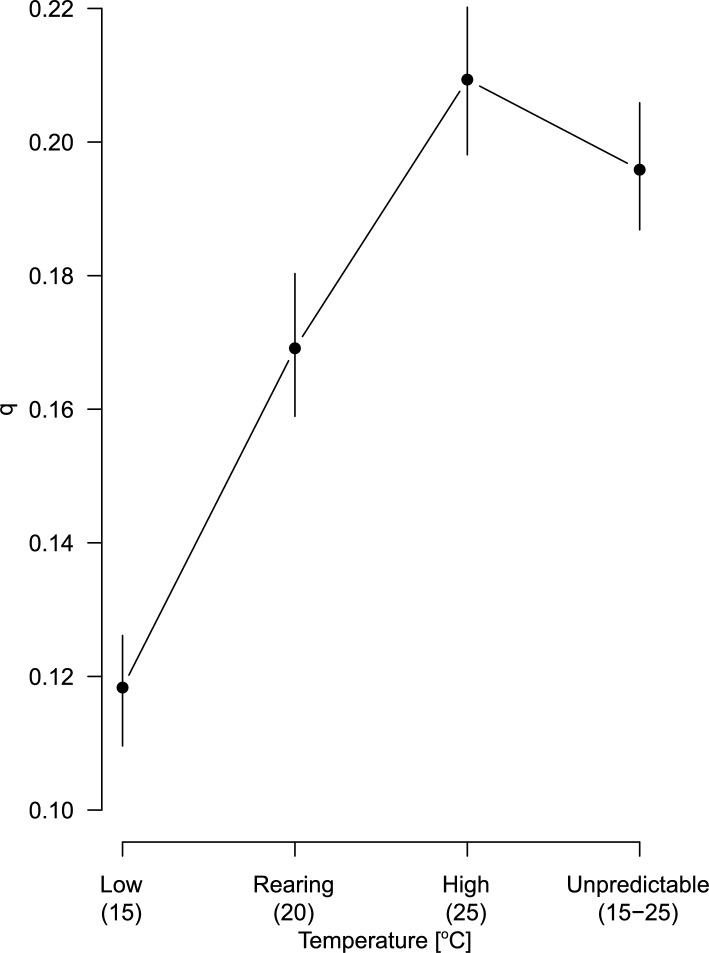
The estimated parameters $\hat{q}$ with their $95\%$ confidence intervals against temperature scenarios.

### Body length growth

**Fig. 2:**
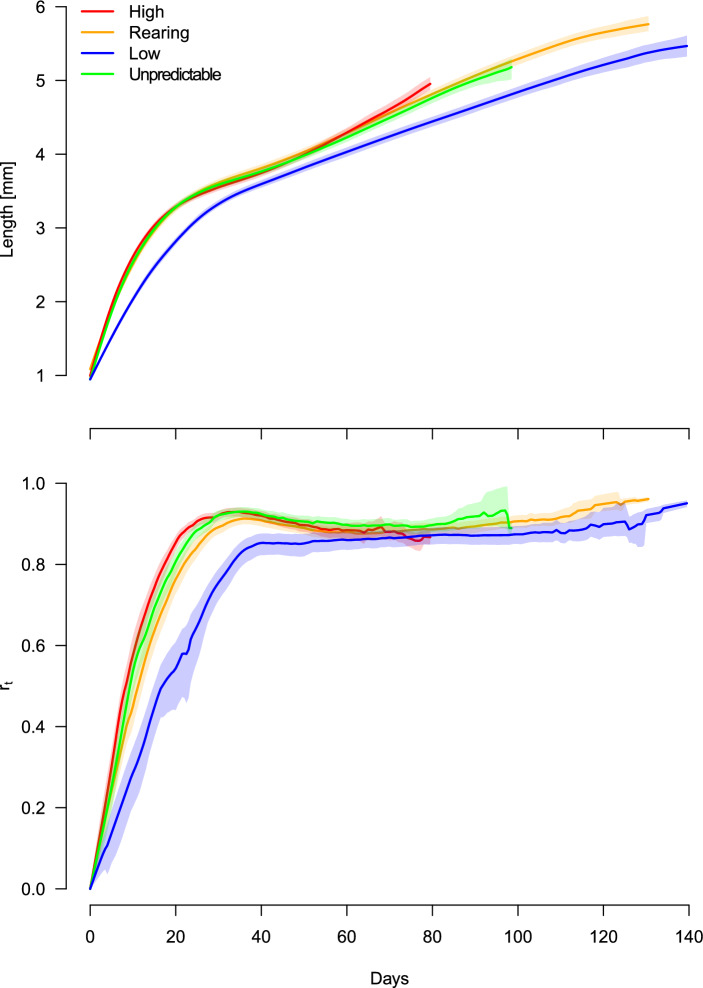
The body length curve and the estimated fecundity changes. Top: the calculated body length growth curve and its $95\%$ confidence envelope for each temperature treatment. Each curve is a numerical solution of model (7), given estimated parameters, $\hat{q}, \hat{\lambda }$ and $\hat{r}_t$. The length of the trajectories differ as the longevity of *D. magna* varies; Bottom: the estimated fecundity investments over time, time-varying parameter $\hat{r}_t$.

**Fig. 3:**
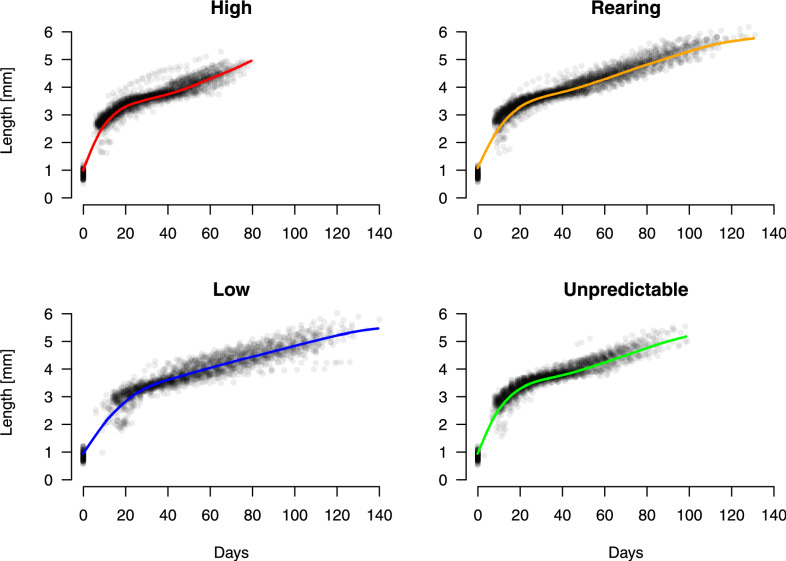
The scatter plots and the estimated growth curves.

The model revealed a significant effect of temperature through the estimated parameter $\hat{q}$ (Table 2 and Fig. 1), equivalent to the energy proportion used for somatic growth. However, there appears to be a slight difference amongst the estimated growth curves, except for that of the low-temperature treatment (Fig. 2, the top panel). The difference in the parameter $\hat{q}$ between the unpredictable- and high-temperature scenarios is relatively moderate. The growth pattern in the individuals exposed to unpredictable temperature variations is parallel to the constant high temperature, with overlapping $95\%$ confidence intervals. Each panel in Fig. 3 illustrates estimated growth curves for the data observed for each temperature treatment. Overall, the curves fit the observations well, and even captured a slight deviation around day 40.

Furthermore, the estimated parameter $\hat{q}$ suggests that *D. magna* in lower temperatures reduce the energy expenditure for growth, demonstrating an exact ascending order along with the low- to high-temperature scenarios (Table 2). Figure 1 highlights a clear linear pattern between the parameter $\hat{q}$ and temperature, except for the unpredictable temperature treatment.

The estimated power coefficient $\hat{\lambda }=0.179$ is assumed to be shared over the different temperature scenarios (A1). The parameter $\lambda$ can easily be converted to the parameter $\alpha$ in the body mass model $(\hbox {1b}^{\prime })$; it is given as $\hat{\alpha }=0.726=2.178/3 \approx 2/3$, which is close to a value widely reported from other studies^13^, when the isometric growth (*i.e.* the cubic weight–length transformation, $k=3$) is assumed.

### Energy allocation between growth and direct reproduction

The estimated time-varying parameters $\hat{r}_t$ highlight variations in relative energy allocation to reproduction across the temperature scenarios (Fig. 2, bottom panel); note that $r_0=0$, implying no energy allocation to reproduction at birth, $t=0$. The discrepancy of these estimated curves appears to be greater for the early life stage (0–40 days) and diminishes in the later stage, as these $\hat{r}_t$ curves approach an asymptote. The constant high-temperature induces rapid gonadic growth with increased relative energy allocation to reproduction within a shorter interval (0–20 days). This response mirrors those in the unpredictable temperature treatment. On the other hand, the individuals allocated to the constant low-temperature treatment take longer (0–35 days) to reach the asymptote. The extent of increase in $\hat{r}_t$ during early life stages becomes faster as temperature increases.

### Direct reproduction

**Fig. 4:**
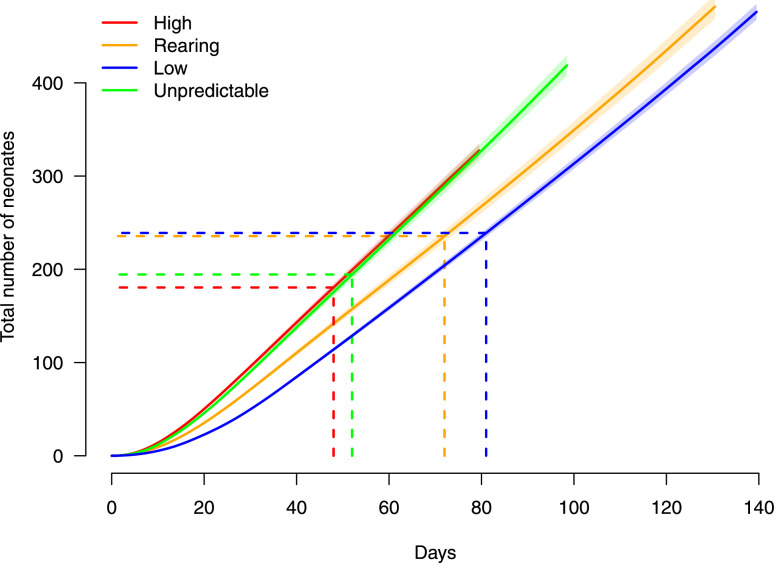
The calculated quantity equivalent to the number of neonates produced for the average lifetime. Each dashed line illustrates the average lifetime (days) $\tau$: 81 (Constant Low), 72 (Constant Rearing), 48 (Constant High) and 52 (Unpredictable Variation). The corresponding number of neonates $\hat{N}_\tau$: 239 (Constant Low), 236 (Constant Rearing), 181 (Constant High) and 194 (Unpredictable Variation).

With the estimated model parameters, namely $\hat{q}, \hat{\lambda }$, and $\hat{r}_t$, Eq. (8) can be matched with the cumulative number of neonates produced by each temperature treatment. Figure 4 illustrates these matched curves for ease of comparison. The average lifetime $\tau$ of the temperature scenarios results in descending order as temperature increases (Table 2). Interestingly, the parameter *q* and the average lifetime $\tau$ are negatively correlated (Table 2). Furthermore, the cumulative number of neonates up to the average lifetime, $\hat{N}_\tau$, is greater for those allocated to the constant low-temperature treatment than the constant high-temperature treatment (Fig. 4 and Table 2).

**Fig. 5:**
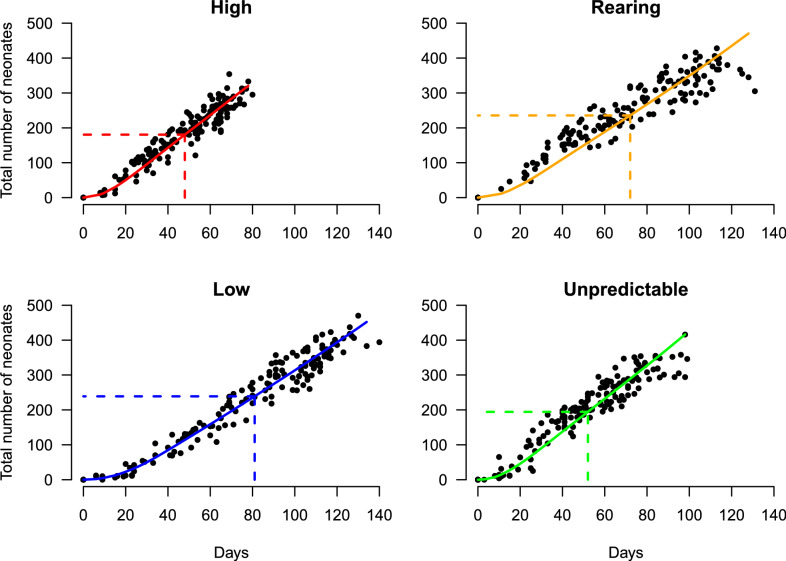
The scatter plots show the number of neonates produced by individuals over the lifetime. The superposed solid line represents the theoretical number of neonates up to days *t*. The dashed line illustrates the average lifetime $\tau$ and the corresponding number of neonates $\hat{N}_\tau$.

The illustrated curves for the cumulative number of neonates in Fig. 5 demonstrate a remarkable agreement with actual observations, indicating that the model provides accurate representation of experiment data. Note that all the model parameters are estimated solely based on the body length data.

## Discussion

We have developed a novel allometric scaling growth model that enables predicting patterns in energy allocation between growth and reproduction across different thermal conditions. With body length data, rather than body mass, this new model broadens its applicability across a range of organisms. As a unique feature of our model, its time-varying parameter, $r_t$, projects instantaneous relative fecundity investment. This parameter delineates how temperature influences the allocation of energy to direct reproduction over a lifetime, capturing age-dependent transitions within unobservable energy distribution mechanisms, and uncovers the interplay between growth and reproduction through a single equation (Eq. 7). The underlying model assumptions (A1–A3) have also been thoroughly evaluated against lifetime neonate data (Fig. 5), thereby ensuring robustness of the model. This novel framework offers valuable insights into the crucial role of energy allocation mechanisms in shaping life-history traits of organisms under realistically varying temperature scenarios, thus revealing the complexity between life-history traits and energy allocation patterns.

The interplay between growth and reproduction has been well-documented under constant temperatures^1^. High mean temperatures are generally associated with increased growth and reproduction rates^3,44^. This pattern is effectively described by our allometric model. Individuals exposed to consistently high temperatures exhibited faster growth with high $\hat{q}$ and produced more neonates early in life as $\hat{r}_t$ illustrated (Table 2, Figure 2). This result provides robustness to our model and aligns with the established expectations of energy allocation between growth and reproduction under consistently higher temperature^45^. Our model, however, shows that when exposed to low-temperature there is a delay in investment in fecundity ($\hat{r}_t$ increased slowly), at the expenses of more energy being allocated to maintenance (Table 2, Fig. 2). This pattern is consistent with the expectation that consistent exposure to below optimal temperatures, energy is prioritised to maintenance at the expenses of reproduction^46^. We did notice a cost of life expectancy in both the unpredictable and constant high temperature scenarios. Under these thermal conditions, individuals produced more neonates during early to mid-life stages but attained shorter lifespan, which resulted in a reduced overall fitness. Greater metabolic demands caused by high temperatures are expected favour a strategy of strong early investment in fecundity at the expenses of reduced lifespan^47,48^. Our results, indicate that *D. magna* adopts a life history strategy analogous to a “live fast, die young” pace-of-life syndrome when exposed to above optimal thermal conditions, a pattern observed in previous studies and hypothesised to evolve in response to such physiological and thermal stressors^49^.

As increased temperature variability intensifies globally^50–52^, organisms face not only warmer averages but also its unpredictability with which becomes a greater challenge to cope^53^. A recent meta-analysis showed limited effect of fluctuating temperatures on biological responses compared to constant temperatures^9^. Our results provide mixed support for this prediction. While individuals exposed to unpredictable variations in temperature produced less neonates at their average lifetime relative to both low and rearing conditions, these effects were not distinct from those observed under the constant high-temperature scenario (Figure 5). Indeed, their growth, reproduction and mortality responses also resemble those of both unpredictable and high-temperature scenarios. This result adds to the discussion regarding whether unpredictable temperature disproportionately impacts fitness cost^38,53^ or not^54^. Several explanations could account for our result. First, individuals under the constant high-temperature scenario were likely to be exposed to their maximum reproductive thermal tolerance and stress^55–57^, thereby always being exposed to thermal stress. On the other hand, individuals exposed to unpredictable temperatures are likely to have encountered periodic optimal thermal conditions. This fluctuation between upper-limit and optimal temperatures may have facilitated a thermal tolerance, thereby reducing some fitness costs associated with unpredictability in temperature^58^. Another possibility is that elevated temperatures impair food-to-energy assimilation efficiency^59^. Our findings confirm the complexity of individual sensitivity responses to thermal variability, which warrants further investigation.

There are some limitations in our study. First, we used the number of neonates at emergence as a proxy for direct reproductive investment. While number and offspring size are considered to be reasonable indicators of fitness and reproductive investment^38,60,61^, indirect reproductive investment such as potential parental care costs can be a crucial component in terms of reproductive investment as a whole^62^, though *Daphnia* provide no parental care. A separate investigation on this energetic component would be worthy as a venue for future research. Further, we note that extrapolations of our model across very-distant species, or extreme size ranges need caution. Despite these limitations, our model provides a clear and robust pattern of the dynamics between energy allocation and life-history traits under ecologically relevant conditions in a keystone species. The allometric nature of our model can be applied to different species to predict their biological rates and traits.

Shifts in fitness-related traits (*e.g.* fecundity, mortality and growth) in response to environmental disruptions—such as increased unpredictability in temperature—bear direct consequences for population dynamics, thus shaping ecosystem stability. Responses in keystone species (such as *Daphnia*) to variability in environmental conditions provide insights into species’ climatic tipping points^63^, which is a critical metric for forecasting the resistance and resilience of the impacted ecosystem^64^. Keystone species play a pivotal role in regulating the balance between primary producers and consumers biomass. For instance, increased *Daphnia* abundance is followed by a decrease in algal blooms, which resulted in improved water quality and increased fish biomass^65^. Disruptions to the life cycle of *Daphnia*, therefore, hold major consequences for the whole ecosystem^66^. The ecological importance of keystone species raises a crucial aspect, how these species respond to environmental unpredictability, which hinders the ability of species to predict future conditions^67–69^. This underlines an urgent need for an improved understanding the extent to which environmental variability shapes life-history traits, leading it into changes at an individual, population, community and ecosystem as a whole. Translating life-history trait responses into energetic responses is, as our model has demonstrated, a promising approach and can address such a gap as a critical first step to a better understanding of processes in species adaptation and resilience, offering great potential for investigating environmental impacts on organisms under emerging changes in climate conditions.

## Supplementary Information

Supplementary Information.

## Data Availability

The datasets analysed during the current study are available in the GitHub, https://github.com/hshimadzu/DaphniaGrowth
