# Supplementary material for: Allometric scale model reveals temperature effects on growth and reproduction in Daphnia magna
Source: Sci Rep. 2025 Aug 22;15:30952. doi: 10.1038/s41598-025-15593-6 (PMC12373993; doi:10.1038/s41598-025-15593-6)
Supplement: Supplementary file 1 — Supplementary Information. [file 41598_2025_15593_MOESM1_ESM.pdf]

Supplementary Information for Allometric scale model reveals temperature effects on growth and reproduction in *Daphnia magna*

Hideyasu Shimadzu[1] **and Miguel Barbosa**[2,3,*]

[1]Department of Data Science, Kitasato University, Kanagawa, Japan
[2]Centre for Biological Diversity, School of Biology, University of St Andrews, Fife, UK
[3]Department of Biology, University of Aveiro, Aveiro, Portugal
[*]mb334@st-andrews.ac.uk

ABSTRACT

Under the unpredictable variation treatment, the temperature fluctuates within different temperature ranges according to three specific time segments every day. From 00:00 to 08:00 and 18:00 to 24:00 (dawn–morning and late afternoon segments), the temperature fluctuates within 15–20°C; from 08:00 to 18:00 (morning–afternoon segment), it randomly varies within 20–25°C. Figure S1 illustrates a seven-day temperature profile during the experiment.

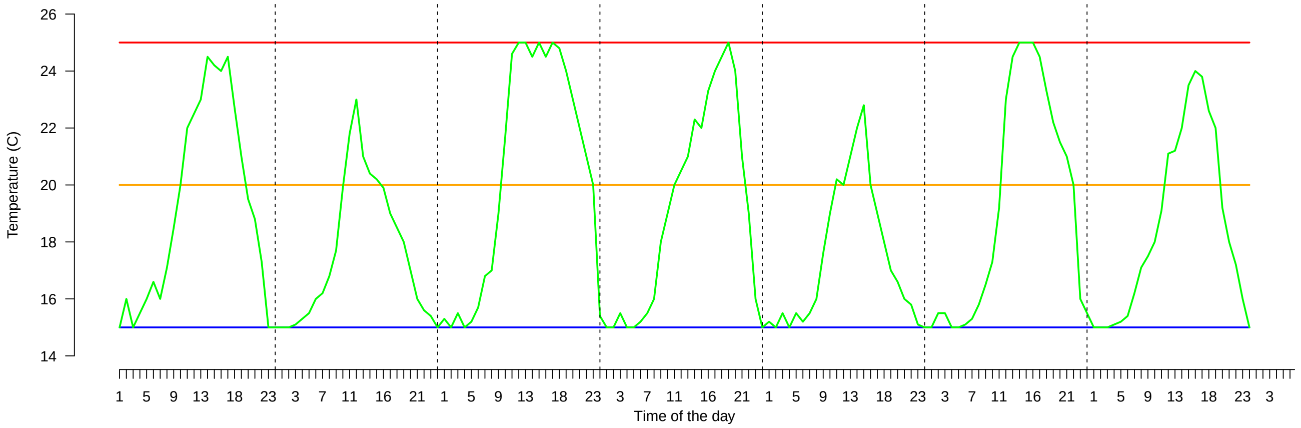

Figure S1. The green line represents the varying temperature profile for a seven-day period. The red, orange, and blue horizontal lines indicate the temperature scenarios: constant high, rearing, and low, respectively.
